# Distinct transcriptional repertoire of the androgen receptor in ETS fusion-negative prostate cancer

**DOI:** 10.1038/s41391-018-0103-4

**Published:** 2018-10-26

**Authors:** Anders E. Berglund, Robert J. Rounbehler, Travis Gerke, Shivanshu Awasthi, Chia-Ho Cheng, Mandeep Takhar, Elai Davicioni, Mohammed Alshalalfa, Nicholas Erho, Eric A. Klein, Stephen J. Freedland, Ashley E. Ross, Edward M. Schaeffer, Bruce J. Trock, Robert B. Den, John L. Cleveland, Jong Y. Park, Jasreman Dhillon, Kosj Yamoah

**Affiliations:** 1 Department of Biostatistics & Bioinformatics, H. Lee Moffitt Cancer Center & Research Institute, Tampa, FL USA; 2 Department of Tumor Biology, H. Lee Moffitt Cancer Center & Research Institute, Tampa, FL USA; 3 Department of Oncological Sciences, University of South Florida, Tampa, FL USA; 4 Department of Cancer Epidemiology, H. Lee Moffitt Cancer Center & Research Institute, Tampa, FL USA; 5 GenomeDx Biosciences Inc, Vancouver, BC Canada; 6 Glickman Urological and Kidney Institute, Cleveland Clinic, Cleveland, OH USA; 7 Department of Surgery, Division of Urology, Center for Integrated Research on Cancer and Lifestyle, Samuel Oschin Comprehensive Cancer Center, Cedars-Sinai Medical Center, Los Angeles, CA USA; 8 Texas Urology Specialists, Dallas, TX USA; 9 Department of Urology, Northwestern University, Chicago, IL USA; 10 Department of Urology, Johns Hopkins, Baltimore, MD USA; 11 Department of Radiation Oncology, Sidney Kimmel Medical College at Thomas Jefferson University, Philadelphia, PA USA; 12 Department of Pathology, H. Lee Moffitt Cancer Center & Research Institute, Tampa, FL USA; 13 Department of Radiation Oncology, H. Lee Moffitt Cancer Center & Research Institute, Tampa, FL USA

**Keywords:** Prostate cancer, Biomarkers

## Abstract

**Background:**

Prostate cancer (PCa) tumors harboring translocations of *ETS* family genes with the androgen responsive *TMPRSS2* gene (ETS+ tumors) provide a robust biomarker for detecting PCa in approximately 70% of patients. ETS+ PCa express high levels of the androgen receptor (AR), yet PCa tumors lacking *ETS* fusions (ETS−) also express AR and demonstrate androgen-regulated growth. In this study, we evaluate the differences in the AR-regulated transcriptomes between ETS+ and ETS− PCa tumors.

**Methods:**

10,608 patient tumors from three independent PCa datasets classified as ETS+ (samples overexpressing ERG or other ETS family members) or ETS− (all other PCa) were analyzed for differential gene expression using false-discovery-rate adjusted methods and gene-set enrichment analysis (GSEA).

**Results:**

Based on the expression of AR-dependent genes and an unsupervised Principal Component Analysis (PCA) model, AR-regulated gene expression alone was able to separate PCa samples into groups based on ETS status in all PCa databases. ETS status distinguished several differentially expressed genes in both TCGA (6.9%) and GRID (6.6%) databases, with 413 genes overlapping in both databases. Importantly, GSEA showed enrichment of distinct androgen-responsive genes in both ETS− and ETS+ tumors, and AR ChIP-seq data identified 131 direct AR-target genes that are regulated in an ETS-specific fashion. Notably, dysregulation of ETS-dependent AR-target genes within the metabolic and non-canonical WNT pathways was associated with clinical outcomes.

**Conclusions:**

ETS status influences the transcriptional repertoire of the AR, and ETS− PCa tumors appear to rely on distinctly different AR-dependent transcriptional programs to drive and sustain tumorigenesis.

## Introduction

The androgen receptor (AR), a nuclear hormone transcription factor, directs the transcription of several genes implicated in the development and progression of prostate cancer (PCa). Given the critical roles AR plays in PCa progression, advanced PCa is often treated with an androgen deprivation therapy (ADT)-based regimen, which depletes the natural ligands of AR, testosterone and dihydrotestosterone. However, several acquired gain-of-function mutations and modifications of the *AR* gene can occur during ADT and these are major mechanisms that drive androgen-independent PCa progression [1]. Accordingly, development and progression of PCa is often studied in the context of AR signaling, yet less is known regarding AR-regulated molecular pathways in PCa progression.

Genomic aberrations in members of the E26 transformation-specific (ETS) family of oncogenic transcription factors (*ERG*, *ETV1*, *ETV4*, *ETV5*, and *FLI1*) are early carcinogenic events found in up to 70% of PCa [2]. *ETS* gene translocations are AR-regulated events, and most frequently this results in overexpression of ETS family genes by placing them under the control of the androgen-responsive promoter *TMPRSS2* [3–5]. Furthermore, ectopic activation of ETS transcription factors directs expression of several biomarkers, including AR-regulated genes [6–10]. Notably, up to 90% of aggressive early-onset PCa tumors appear to be ERG+, and ETS+ tumors have elevated AR expression and somatic alterations that are androgen-driven, including the expression of *TMPRSS2-ETS* fusions [8]. Finally, molecular and biological studies of ETS+ tumors have revealed that sites of DNA damage are often located near AR binding sites [11], that ERG and AR have overlapping transcription targets, and that ERG inhibits AR-directed differentiation of prostate epithelial cells [12].

Despite the fact AR is also expressed in PCa tumors lacking ETS fusions (ETS−) [13], the role of AR activity in ETS− tumors is poorly understood, and there are also very little data regarding the landscape of somatic alteration of ETS− tumors. Here we report striking differences in the AR transcriptional programs of ETS+ versus ETS− PCa that establish marked differences in the biology of these tumors.

## Results

### AR-regulated genes differentiate ETS− and ETS+ PCa tumors

**Fig. 1:**
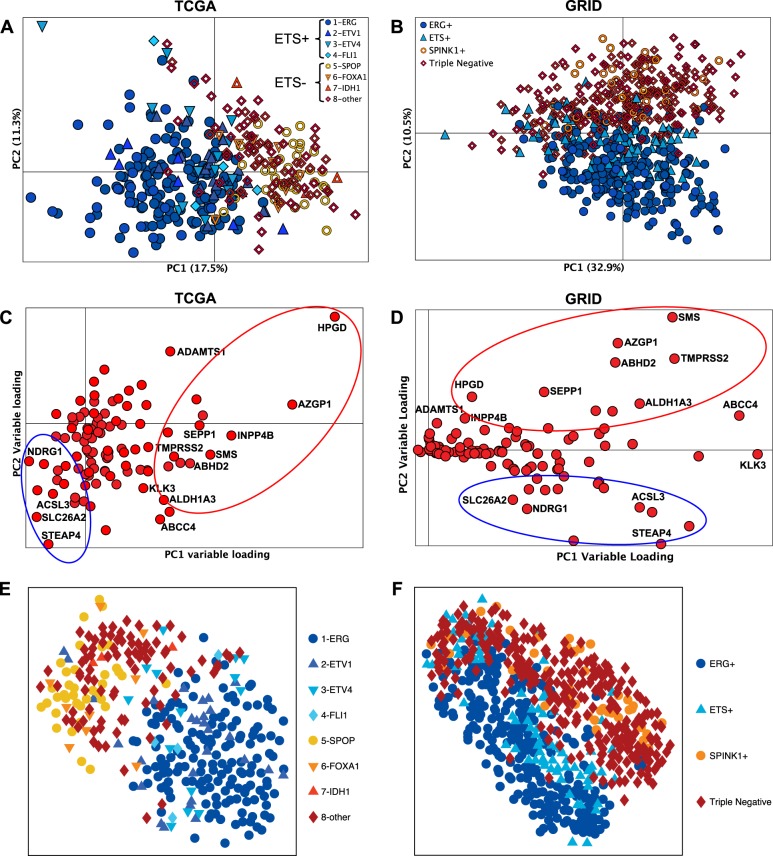
AR-regulated genes discriminate tumors based on ETS status. An unsupervised PCA model of 101 AR-regulated genes affirms the distinct AR signatures of ETS+ and the ETS− samples in the first principal component for the TCGA RNA-seq (**a**) and in the second principal component for the GRID microarray profiling (**b**) PCa datasets. **c** and **d** show the relative contribution of the individual AR-regulated genes to the PCA models in **a** and **b**, respectively. The t-SNE model shows similar results to the PCA model for the TCGA (**e**) and the GRID (**f**) dataset, where there is a clear separation between the ETS− and ETS+ samples. The results also indicate that the molecular subtypes used for ETS+ (1-ERG, 2-ETV1, 3-ETV4, 4-FLI1) and ETS− (5-SPOP, 6-FOXA1, 7-IDH1, 8-other) fall into the correct ETS category

ETS status (ETS+ or ETS−) was determined for all PCa samples within the TCGA, GRID, and GRID-prospective datasets (Table 1). Clinical, demographic, and pathological characteristics of patients within the TCGA and GRID cohorts stratified by ETS status is shown in Supplementary Table S1. An unsupervised principal component analyses (PCA) was performed in the TCGA (Fig. 1a) and GRID (Fig. 1b) dataset. PCA showed a robust separation of ETS+ and ETS− tumors based on their expression profile of genes present in the HALLMARK_ANDROGEN_RESPONSE gene list, which are well-validated AR regulated genes (Fig. 1a, b). The loading plots for the PCA models indicate the individual AR-dependent genes that are differentially expressed in both the TCGA and GRID datasets (Fig. 1c, d, respectively). Finally, a similar clustering of ETS+ versus ETS− samples based on AR-dependent genes was manifest using a second unsupervised method for sample clustering, *t*-SNE (*t*-distributed stochastic neighbor embedding) (Fig. 1e, f). Thus, known AR-regulated genes are differentially expressed in ETS− prostate tumors compared to ETS+ tumors, suggesting AR activates distinct biological pathways in ETS− versus ETS+ PCa.

**Table. 1:** ETS status of PCa tumors in TCGA and GRID datasets

ETS subtypes	TCGA (*N* = 333)	GRID (*N* = 635)	GRID-prospective (*N* = 9640)
ETS−	*N* = 135	*N* = 309	*N* = 4757
Variants	SPOP	SPINK1+	SPINK1+
	FOXA1	Triple negative	Triple negative
	IDH1		Other
	Others		
ETS+	*N* = 198	*N* = 326	*N* = 4883
Variants	ERG	ERG+	ERG+
	ETV1	ETS+	ETV1
	ETV4		ETV4
	FLI1		FLI1

*TCGA* cancer genome atlas research network; *GRID* genomic resource information database; *Triple negative* ERG−, ETS− and SPINK1−

### Unique androgen response genes are differentially upregulated in ETS− and ETS+ prostate tumors

**Fig. 2:**
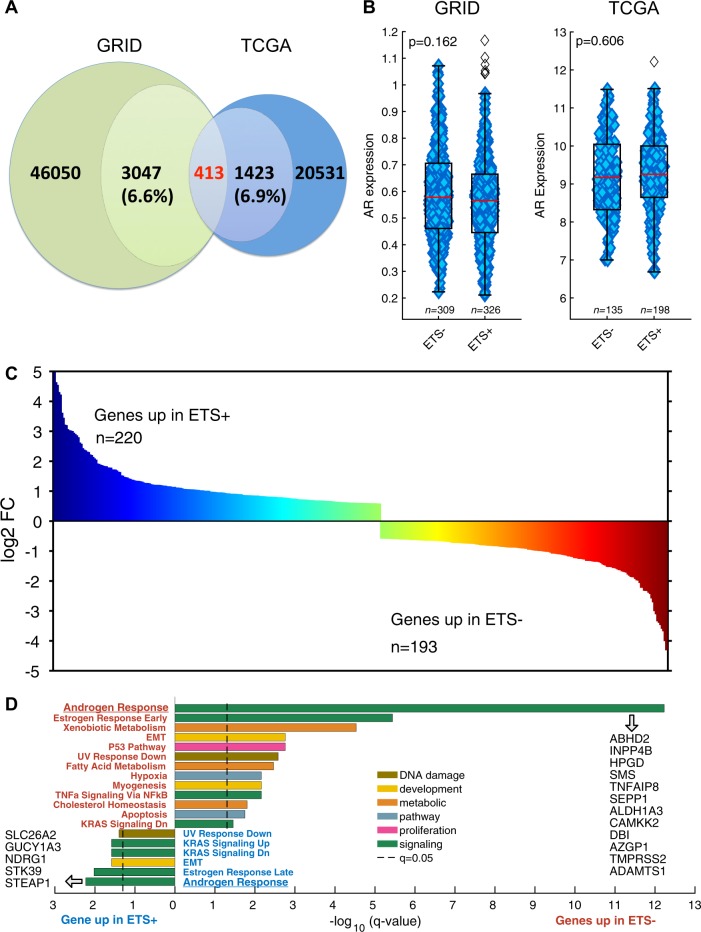
Genes differentially expressed in ETS+ versus ETS− PCa tumors. **a** The GRID dataset has 46050 genes with 3047 (6.6%) being differentially expressed when comparing ETS+ vs. ETS− PCa. The TCGA dataset has 20531 genes with 1423 (6.9%) being differentially expressed based on ETS status. Using a false-discovery-rate adjusted (*q* < 0.05) Mann–Whitney U test and a fold-change cut-off of 0.585 (TCGA) and 0.05 (GRID), 413 differentially expressed genes based on ETS status were defined in both PCa databases. **b** There is no significant difference in AR expression between ETS− and ETS+ tumors in either TCGA or GRID. The 413 significant differentially expressed genes (**c**) in the TCGA and GRID PCa based on ETS status were analyzed by GSEA and the HALLMARK gene sets [43]. Significant gene sets (**d**) overexpressed in ETS+ are shown at the top and those gene sets for ETS− shown below. Androgen response was the top-ranked gene set for both ETS+ and ETS− significantly overexpressed genes

To investigate if discrete biological pathways are upregulated in ETS− prostate tumors compared to ETS+ prostate tumors, we identified the genes that are differentially expressed between the ETS+ and ETS− cohorts in the TCGA and GRID dataset. This analysis found 1423 (Supplementary Table S2) genes in the TCGA dataset (6.9% of genes analyzed) and 3047 (Supplementary Table S3) genes in the GRID dataset (6.6% of genes analyzed) to be differentially expressed between ETS− and ETS+ tumors. Further, 413 genes were identified as differentially expressed in the two ETS groups in both the TCGA and GRID datasets (Fig. 2a, Supplementary Table S4), where 220 genes were overexpressed in ETS+ cases and 193 genes were overexpressed in ETS− cases. These 413 genes were validated as being differentially expressed based on ETS status in a third independent, non-overlapping cohort, specifically the prospective GRID cohort (GRID-prospective) that is comprised of 9640 PCa patient samples. This analysis established a strong correlation in the expression of these genes between the TCGA dataset, where expression was measured by RNA sequencing, and the GRID-prospective dataset, where expression was measured using a genome-wide microarray platform (*r* > 0.8; Supplementary Fig. S1A). This correlation was even more robust when comparing the GRID and GRID-prospective datasets (*r* ≥ 0.9; Supplementary Fig. S1B), which both used the same microarray platform for gene expression analysis. Percentage tumor purity was also similar between ETS− and ETS+ tumors in both TCGA and GRID databases (Supplementary Fig. S1C and D).

To determine which biological pathways, based on the Hallmark Gene Sets, are over-represented in the 413 genes that are up-regulated in either of the two ETS groups (Fig. 2c), gene-set enrichment analysis (GSEA) was performed. Intriguingly, GSEA revealed that independent, non-overlapping sets of androgen response genes are overexpressed in both ETS− and ETS+ tumors (Fig. 2d), suggesting ETS status alters the repertoire of androgen-regulated genes in PCa. Furthermore, many of the genes upregulated in ETS− tumors are involved in metabolic, p53 or hypoxia pathways, whereas this was not the case in ETS+ tumors that showed more prominent association with the epithelial mesenchymal transition (EMT) and KRAS signaling pathways (Fig. 2). Importantly, AR expression by itself is not dependent on ETS status as shown in Fig. 2b. Individual genes enriched in corresponding pathways are provided in Supplementary Fig. S2 A-B. Thus, ETS− and ETS+ prostate tumors both show comparable expression of AR, yet demonstrate unique AR-dependent alterations in biological pathways that drive tumorigenesis.

### AR directs distinct transcriptional programs in PCa based on ETS status

**Fig. 3:**
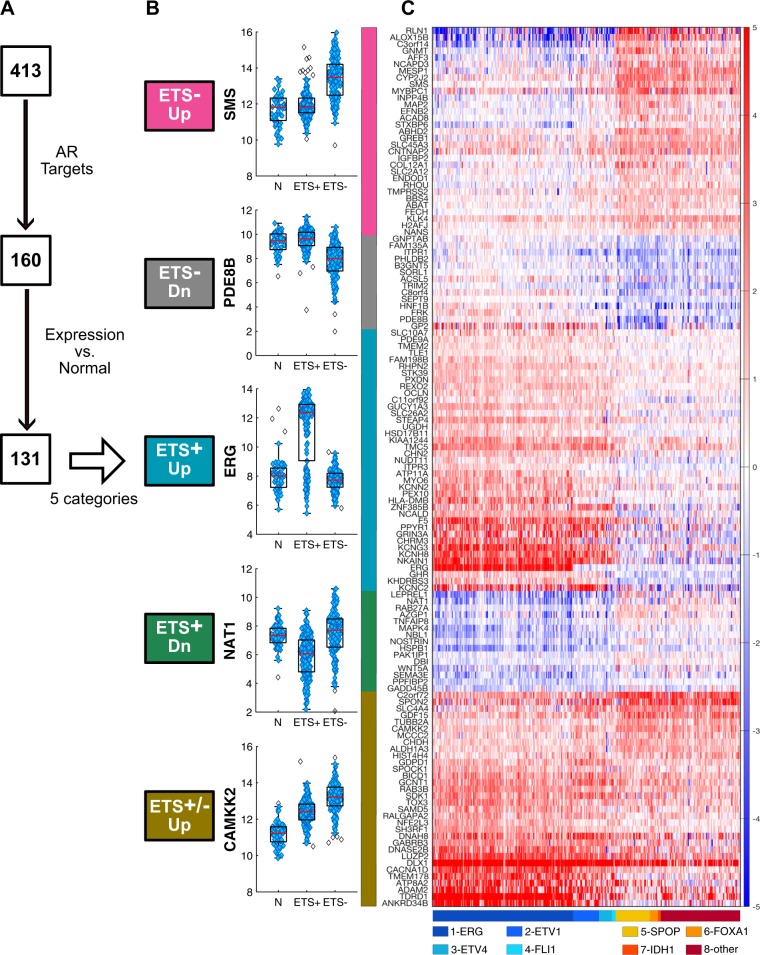
Distinct direct AR target genes are regulated in ETS+ and ETS− PCa tumors. **a** Schematic of pipeline used to define 5 categories from the 131 direct AR transcription targets in ETS+ and ETS− PCa tumors. **b** Examples of genes whose expression is significantly different in adjacent normal tissue (N) in ETS+ PCa or ETS− PCa. **c** Heatmap of differentially expressed direct AR target genes in ETS- Up pink, ETS- Down gray, ETS+ Up blue, ETS+ Down green, and that are upregulated in both ETS+ and ETS− PCa (brown). Each row/gene is normalized to median expression in adjacent normal tissue. The 8 subtypes of ETS+ and ETS− PCa, as defined by their expression of *ERG*, *ATV1, ETV4, FLI1, SPOP, FOXA1, IDH1* and “other” are shown beneath the heatmap

Since ETS− and ETS+ prostate tumors differentially expressed distinct sets of androgen response pathway genes, we assessed if the AR directs distinct transcriptional programs in PCa based on ETS status. To do so, we queried the 220 genes overexpressed in ETS+ cases and 193 genes overexpressed in ETS− cases in three publicly available AR ChIP-seq datasets [14], which defined direct AR targets as those genes that display AR binding to an androgen response element within 25 kb of the target gene’s promoter, in several PCa cell lines [12, 14, 15], primary prostate tumor tissues [15], and metastatic PCa [16]. These analyses revealed that a total of 160 of these genes were not only direct AR target genes but were also regulated in an ETS-specific manner (Fig. 3a). To further analyze these 160 AR targets, their relative levels of expression in adjacent non-tumor prostate sample was compared to their expression in ETS− tumor samples and ETS+ tumor samples using the TCGA dataset. Using the subtractive genomics method, we removed 29 AR target genes that were not significantly different between the PCa tumors and the adjacent normal prostate tissues. The remaining 131 AR target genes belonged to five distinct categories based on ETS status: (1) ETS− Up (genes upregulated only in ETS− tumors), (2) ETS− Dn (genes downregulated only in ETS− tumors) (3) ETS+ Up (genes upregulated only in ETS+ tumors), (4) ETS+ Dn (genes downregulated only in ETS+ tumors), and (5) ETS−/ETS+ Up (genes upregulated in both ETS− and ETS+ tumors). As shown in Fig. 3b, *SMS*, *PDE8B*, *ERG*, *NAT1* and *CAMKK2* are representative genes identified for each of these five categories, which were clearly evident in a clustering analyses of the 131 direct AR target genes that are differentially expressed based on ETS status in PCa, as well as by the relevant subtypes that have been identified for ETS+ and ETS− prostate tumors (Fig. 3c). Strikingly, all 131 AR target genes were validated by an independent subtractive genomics and clustering analyses performed in 9640 samples from the Microarray-based GRID-prospective dataset (Table 1; Supplementary Fig. S3)

### AR transcriptional programming in prostate tumor is ETS-dependent

**Fig. 4:**
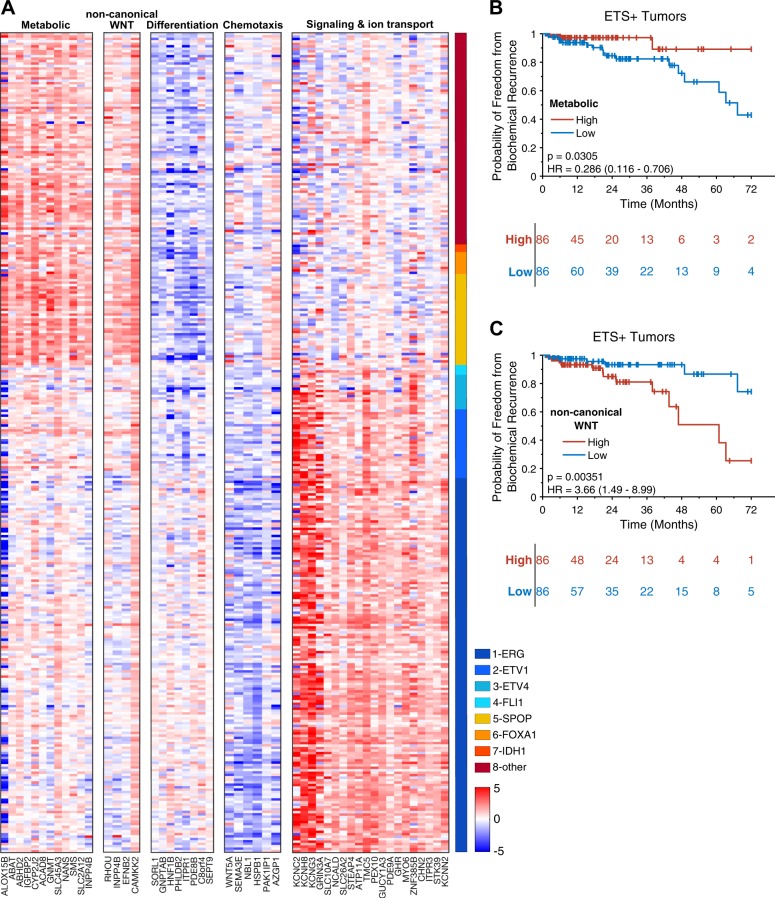
Specific AR target pathways show ETS status and BCR dependency. Gene ontology analysis and literature searches revealed that among ETS status-dependent AR target genes, metabolic pathway genes and non-canonical WNT pathway genes are up-regulated in ETS− tumors, whereas signaling and ion transport pathway genes are up-regulated in ETS+ tumors (**a**). Kaplan–Meier curves for metabolic (**b**) and non-canonical WNT (**c**) genes show significance to BCR in ETS+ tumors

To identify biological pathways that may be involved in ETS-dependent PCa development, extensive literature searches coupled with gene ontology analysis was performed using GO biological process gene sets on the five distinct ETS-dependent AR target genes categories (Supplementary Table S5). Four of the five categories of ETS-dependent PCa AR target genes were enriched for specific biological pathways (Supplementary Table S5). These five pathways (metabolic, non-canonical WNT, differentiation, chemotaxis, and signaling & ion transport) with their corresponding genes are presented in Fig. 4a as a heatmap, where the expression values were normalized using median expression in normal adjacent tissue. This heatmap highlighted the up-regulation of metabolic pathway genes and the non-canonical WNT pathway, and downregulation of genes involved in cellular differentiation, in ETS− tumors. Conversely, ETS+ tumors have reduced expression of genes that suppress chemotaxis and cell motility and an upregulation of genes involved in signal transduction and ion transport (Fig. 4). Thus, there are profound differences in the AR repertoire of ETS+ and ETS− PCa that suggest marked differences in the biology of these tumors.

To test if these gene signature pathways were related to biochemical recurrence (BCR) a PCA model was applied to data from the TCGA cohort. The first principal component (PC1) of the non-canonical PCA model explains 55.9% of the variation, which is better than 99.6% of all random gene PCA models. The ration of PC1/PC2 is 3.3 which is better than 99.99% of the random gene model. The corresponding results for the metabolic pathway is 51.5% explained the variance for PC1 (better than 99.99% of the random models), and with a PC1/PC2 ratio of 5.9 (better than 99.99 % of the random models). These results indicate that the generated gene signatures are robust and better than random gene signatures [17, 18]. Notablty, two of these pathways, the non-canonical WNT pathway and the metabolic pathway genes, showed a significant association with BCR only in the ETS+ cohort. Specifically, low expression of metabolic pathway genes and high expression of non-canonical WNT pathway genes was associated with worse biochemical recurrence (Fig. 4b, c); the corresponding results for the ETS− tumors are shown in supplementary figure S4.

## Discussion

The systematic analysis of the transcriptional landscape of PCa tumors based on ETS status reported herein reveals important insights into the biology of ETS+ versus of ETS− tumors, and establishes that the repertoire of AR target genes is dramatically affected by ETS fusion oncoproteins. For example, GSEA analysis demonstrated that metabolic pathway genes, including genes involved in xenobiotic and fatty acid metabolism, were preferentially enriched in ETS− but not in ETS+ PCa tumors. Further, although ETS+ and ETS− PCa are both androgen dependent, different sets of androgen response genes are enriched based on ETS status, and analyses of previously published AR ChIP-seq datasets revealed that AR target genes are dysregulated in uniquely different ways in ETS− versus ETS+ tumors. These findings explain the apparently conflicting reports regarding AR activity in ETS− PCa [8], where AR target genes that are dysregulated in ETS− tumors drive metabolism and suppress differentiation. Thus, ETS− tumors have comparable AR expression when compared to ETS+ tumors (as shown in Fig. 2b), but rather utilize distinctly different AR-dependent transcriptional programs to sustain tumor growth and survival.

In an in-depth analysis of clinically relevant ETS-dependent, AR-regulated biological pathways we have identified 5 distinct pathways including the metabolic, non-canonical WNT, differentiation, chemotaxis, and signal transduction and ion transport. ETS+ tumors showed reduced levels of AR target genes within the metabolic pathway that drive polyamine synthesis [19–21], fatty acid and arachidonic acid metabolism [22–24], and phosphoinositol metabolism [25, 26], non-canonical WNT, and chemotaxis pathways [10], and increased expression in differentiation and signal transduction and ion channel transport pathways. The reverse scenario was manifest in ETS− tumors.

Notably, AR regulates the expression of genes involved in polyamine biosynthesis such as spermine synthase (*SMS*) and Glycine-N-methyltransferase (*GNMT*), an enzyme involved in S-adenosyl-methionine (*SAM*) homeostasis. These findings are in accord with those showing that androgen tightly regulates polyamine biosynthesis via control of ornithine decarboxylase (*ODC1*) and SAM-decarboxylase (*AMD1*) [21, 27], and magnetic resonance spectroscopy analyses of clinical samples have suggested spermine as a biomarker for the malignant behavior of PCa [28].

The expression analyses reveal that AR coordinately induces targets involved in arachidonic acid metabolism in both ETS− and ETS+ PCa tumors. First, it is interesting that glycerol and arachidonic acid produced via *ABDH2*, promote prostate tumor growth [23]. Indeed, immunohistochemical analysis of tumor specimens has shown a positive correlation of *ABHD2* levels with high Gleason score, pathological nodal stage, low cancer-specific survival rates, and a resistance to docetaxel-based chemotherapy [23]. Second, *ALOX15B* (arachidonate 15-lipoxygenase type II) known to metabolize arachidonic acid to fatty acid hydroperoxides, is selectively regulated in PCa in an ETS-dependent fashion [29]. Finally, the expression of *CYP2J2*, which promotes tumor cell growth by converting arachidonic acid to epoxyeicosatrienoic acids [24], is also up-regulated in ETS− tumors. Thus, given several other interesting metabolic targets are manifest in both ETS− and ETS+ PCa tumors (Supplementary Fig. S2A-B; Table S5), targeting arachidonate metabolism may represent a particularly attractive therapeutic vulnerability for these tumors.

Of note, our analyses revealed that the WNT pathway is also up-regulated in ETS− PCa. Heretofore WNT/β-catenin signaling has been implicated in PCa tumor cell self-renewal, pathogenesis, and aggressive disease [30], and activated WNT/β-catenin signaling has been described to be among the most highly enriched pathways in ERG overexpressing PCa tumors [31]. However, our analyses also revealed ETS-dependent dysregulation of several AR targets within non-canonical WNT pathway genes, including *RHOU*, *INPP4B*, *EFNB2*, and *CAMKK2* (Fig. 4; Supplementary Table S5;). These findings suggest distinct roles for WNT signaling in driving ETS+ versus ETS− PCa, and further underscore the need for developing alternative strategies for treating these tumor types. Interestingly, our analyses also revealed that reduced expression of metabolic pathway genes and increased expression of non-canonical WNT pathway genes is associated with worse biochemical recurrence. These clinically relevant observations were only evidence in ETS+ PCa tumors (Fig. 4b, c) but not in ETS− tumors (Supplementary Fig, S4). Caveats are that these differences may be due to lower numbers of ETS− tumors within the TCGA cohort and the relatively short median follow up time of 24 months. Alternatively, other yet unidentified pathways may be more clinically relevant among ETS− tumors.

The findings reported herein also have importance regarding health disparities in African American men (AAM), as ETS fusion events only occur in a minority of these prostate tumors [32–36]. Similarly, the proportion of AAM within the TCGA and GRID datasets used for the analyses in this current study had predominantly ETS− tumors (Supplementary Table S1). Our findings further emphasizes the lack of the clinical utility of the ETS+ status as a biomarker for PCa in AAM. While in-depth expression analyses are needed in this at-risk population to support this notion, we were unable to perform these studies due to insufficient number of AAM cases within each of the ETS subgroups.

In conclusion, this study identified distinct androgen-responsive genes in both ETS− and ETS+ prostate tumors, and validated 131 AR-target genes that are regulated in an ETS-specific fashion. Importantly, AR-target genes in ETS− tumors were involved in metabolic and non-canonical WNT pathways, thus reclassifying these genes for use in targeted therapy discovery.

## Methods

### Prostate cancer tumor samples and microarray data

A total of 10,608 radical prostatectomy (RP) tumor expression profiles were used in this analysis. RNA-sequencing (RNAseqV2) gene expression data from the TCGA data portal for PCa and adjacent normal prostate tissue samples for 333 primary PCa tumors and associated clinical information, including molecular subtypes, was retrieved from the TCGA PRAD333 study [3, 37]. Retrospective and prospective microarray gene expression data and associated clinical information, including molecular subtypes was retrieved from Decipher GRID registry (NCT02609269). The retrospective samples (GRID) were from a matched cohort of African American (AAM) and European American (EAM) men microarray data from a previously published study [38, 39]. The prospective GRID cohort (GRID-prospective) was from clinical use of the Decipher test (GenomeDx Biosciences Laboratory, San Diego, CA). Samples from the GRID and GRID-prospective cohorts are non-overlapping and were analyzed using the same Affymetrix microarray platform. The SCAN algorithm was used for individual patient profile pre-processing and normalization [40]. COMBAT was used for debatching [41].

### ETS expression profiling and molecular characterization

ETS+ status was assigned to samples that express high levels of *ERG* and the ETS family members *ETV1-5* and *FLI1*. All other tumors were assigned as ETS−. For TCGA the following molecular subgroups were included in the ETS+ group: 1-*ERG*, 2-*ETV1*, 3-*ETV2*, 4-*FLI1*, and for the ETS− group: 5-*SPOP*, 6-*FOXA1*, 7-*IDH1*, 8-other, which were retrieved from the TCGA PRAD33 study [3]. The ETS status for GRID was determined as previously described for microarray-based ETS+ (*ERG* or *ETV1/4/5*) subtyping [42]. For both TCGA and GRID the expression levels and cut-points used for the molecular subtyping for ETS+ and ETS− status were pre-specified in the datasets used for this analysis [3, 42].

AR targets genes were defined as those genes that were located within 25 kb downstream of an established AR binding site, based on publicly available AR ChiP-seq data from the studies of Massie et al. [14], Sharma et al. [16] and Pomerantz et al. [15].

### Statistical analysis

Principal component analysis (PCA) was performed using 101 androgen response genes as defined in the HALLMARK_ANDROGEN_RESPONSE gene-set [43]. The PCA models were calculated using Evince (Prediktera AB, Sweden). *t*-SNE (*t*-distributed stochastic neighbor embedding) was performed in MATLAB using a perplexity value of 30. All pairwise comparisons were performed using a two-tailed Mann–Whitney U test. Multiple testing was adjusted using FDR (*q* < 0.05). Fold change was also used to define significantly expressed genes (log2 FC > 0.585 for TCGA using RNAseq-based normalization and log2 FC > 0.05 for GRID using SCAN normalization [40]. All statistical tests were performed using MATLAB R2017a (The MathWorks, Inc.). Gene set enrichment analysis (GSEA), using the molecular signatures database (MSigDB), was used to evaluate biologic pathway differences based on ETS status [44]. The HALLMARK [43] and the GO biological process gene sets were used for the GSEA analysis. Heatmaps (Fig. 3c and Fig. 4a) were generated as follows: samples and genes were ordered based on input-order, and for each gene the median expression in normal adjacent tissue was subtracted. All heatmaps were generated in MATLAB R2017a (The MathWorks, Inc.) and R version 3.3.3. The pathways shown in Fig. 4 were derived using gene ontology (GO) annotation and manual literature searches of the 131 selected genes (listed in Supplementary Table S5). The first PCA component was used to summarize gene sets (Fig. 4) and validated to ensure the robustness of the PCA model [17, 18]. Median cut was used to define high and low groups in the BCR analysis. Kaplan−Meier curves and log rank test was performed using MatSurv (github.com/aebergl/MatSurv). This study has been approved by the Institutional Review Board.

## Electronic supplementary material

Supplementary Figure and Table Legends

Supplementary Figure S1

Supplementary Figure S2

Supplementary Figure S3

Supplementary Figure S4

Table S1

Table S2

Table S3

Table S4

Table S5
