# Supplementary material for: Distinct transcriptional repertoire of the androgen receptor in ETS fusion-negative prostate cancer
Source: Prostate Cancer Prostatic Dis. 2018 Oct 26;22(2):292–302. doi: 10.1038/s41391-018-0103-4 (PMC6760558; doi:10.1038/s41391-018-0103-4)
Supplement: Supplementary file 1 — Supplementary Figure and Table Legends [file 41391_2018_103_MOESM1_ESM.docx]

**Supplementary Figure S1**

**Correlation between platforms and tumor purity. A,** Correlation of the fold change of ETS-regulated genes in PCa between the TCGA dataset and the GRID-prospective dataset. **C**, Correlation of the fold change of ETS-regulated genes in PCa between the GRID and the GRID-prospective datasets, which used the same microarray platform for expression analysis. The color indicates relative sample density. There is a small difference in tumor purity between ETS- and ETS+ tumors. The median difference in tumor purity between ETS- and ETS+ tumors is only 2.17% for GRID (**C**) and 1.7% for TCGA (**D**).

**Supplementary Figure S2**

**Individual genes from GSEA analysis**. The individual overexpressed genes for each of thje Hallmark Gene-Sets are shown in **A** for ETS+ and in **B** for ETS- PCa.

**Supplementary Figure S3**

**Validation heatmap of 131 ETS-dependent AR-target genes in the GRID-prospective samples.** Each row/gene is normalized to median gene expression from non-adjacent normal tissue. The 4 subtypes of ETS+ and ETS- PCa, as defined by their expression of ERG, ETV1, ETV4, FLI1, SPINK1, Triple Negative (ERG-, ETS- and SPINK1-), and “other” (shown beneath the heatmap).

**Supplementary Figure S4**

**BCR in ETS- tumors**. Kaplan-Meier curves for Metabolic (A) and non-canonical WNT (B) genes show no significance to BCR in the ETS- cohort.

**Supplementary Table S1**

Clinical, demographic, and pathological characteristics - TCGA and GRID cohorts

**Supplementary Table S2.**

1432 Genes that are significantly differentially expressed between ETS- and ETS+ PCa tumors in the TCGA dataset .

**Supplementary Table S3.**

3047 Genes that are significantly differentially expressed between ETS- and ETS+ PCa tumors in GRID dataset.

**Supplementary Table S4.**

ETS dependent genes that are differentially expressed in both the TCGA and GRID datasets.

**Supplementary Table S5**

**Five categories of ETS dependent AR target genes**. Direct AR target genes that are differentially regulated in ETS- and ETS+ PCa tumors are enriched for distinct AR-dependent pathways, including metabolic, differentiation, chemotaxis and signal transduction.
