# Supplementary figures and images for: Distinct transcriptional repertoire of the androgen receptor in ETS fusion-negative prostate cancer
Source: Prostate Cancer Prostatic Dis. 2018 Oct 26;22(2):292–302. doi: 10.1038/s41391-018-0103-4 (PMC6760558; doi:10.1038/s41391-018-0103-4)

# Supplementary Figure S1

**A**

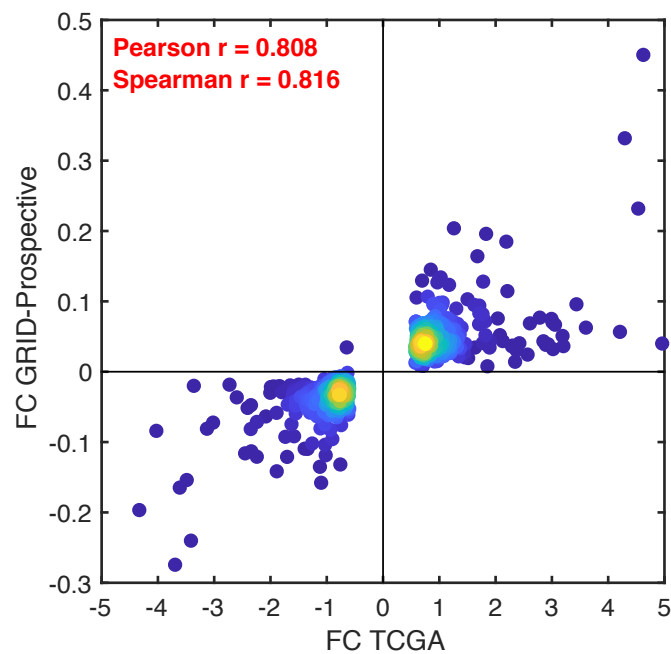

**B**

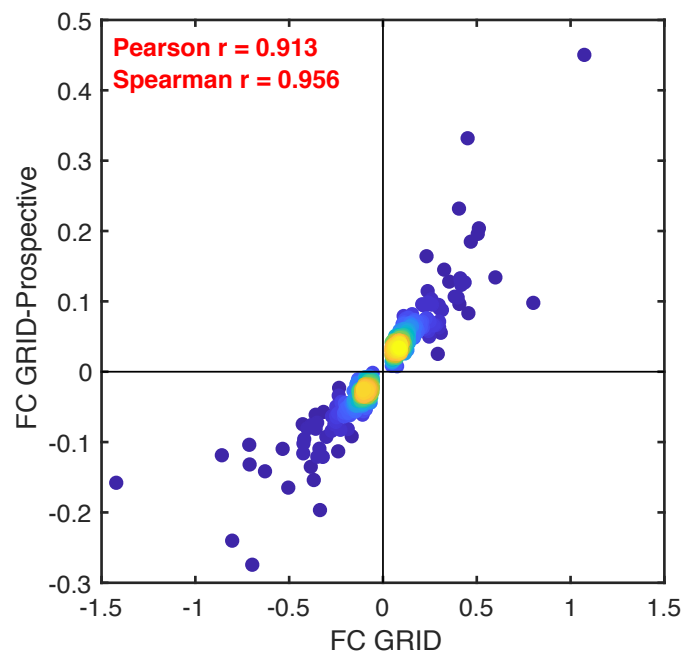

**C**

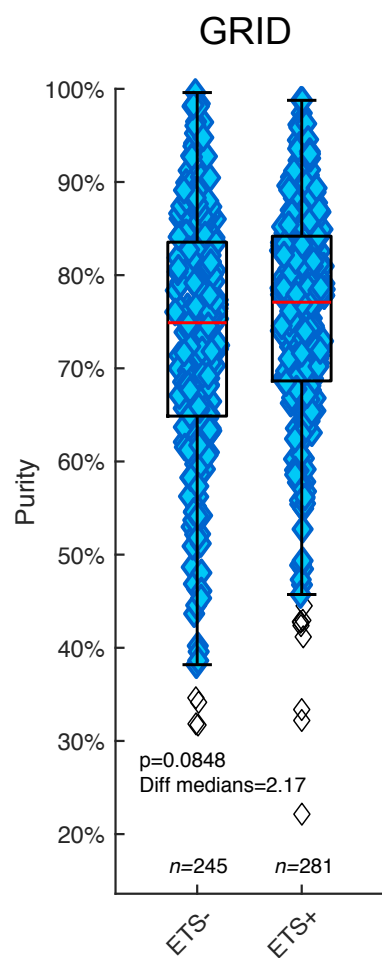

**D**

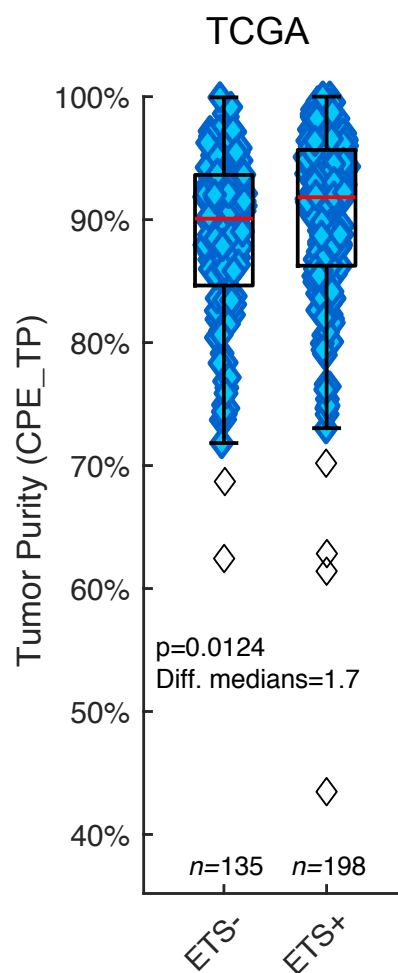

Supplement: Supplementary file 2 — Supplementary Figure S1 [file 41391_2018_103_MOESM2_ESM.pdf]

## Supplementary Figure S2

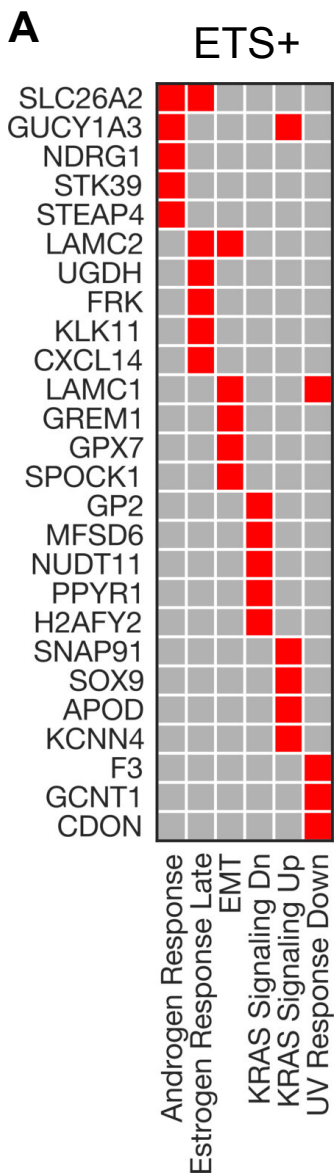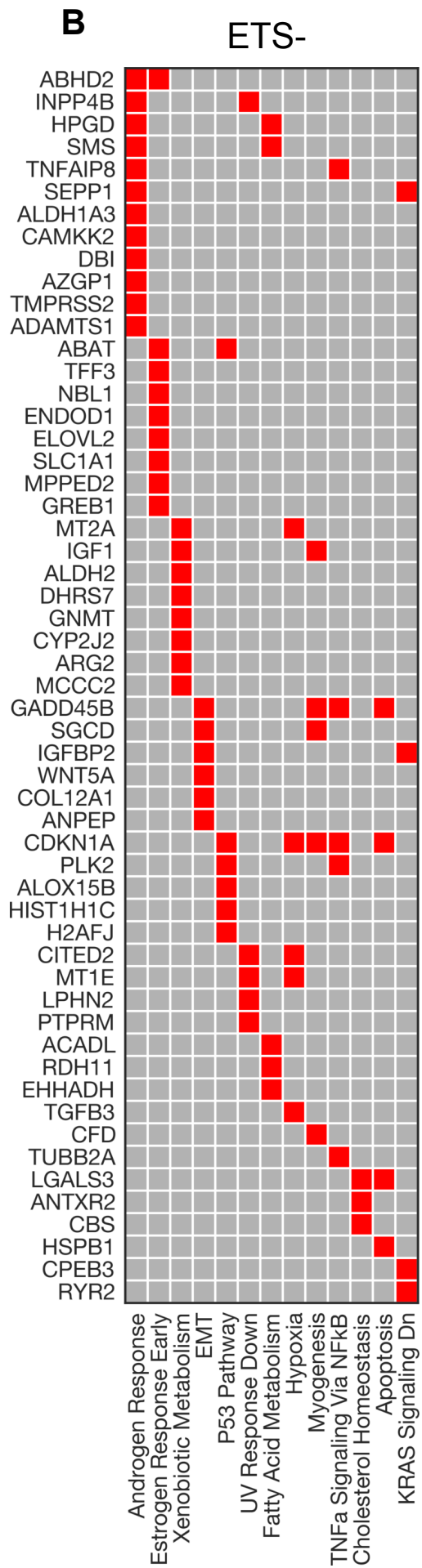

Supplement: Supplementary file 3 — Supplementary Figure S2 [file 41391_2018_103_MOESM3_ESM.pdf]

Supplementary Figure S3

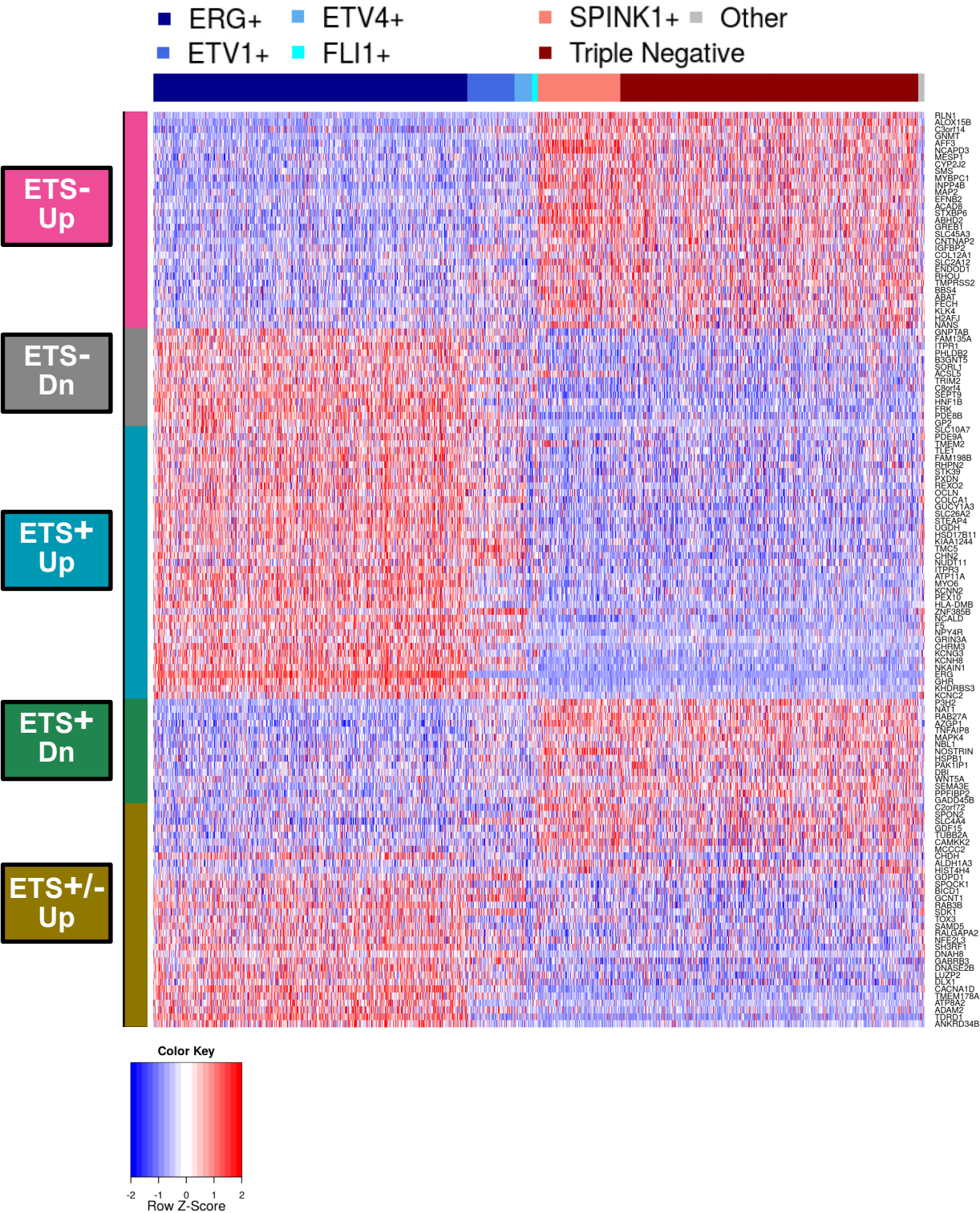

Supplement: Supplementary file 4 — Supplementary Figure S3 [file 41391_2018_103_MOESM4_ESM.pdf]

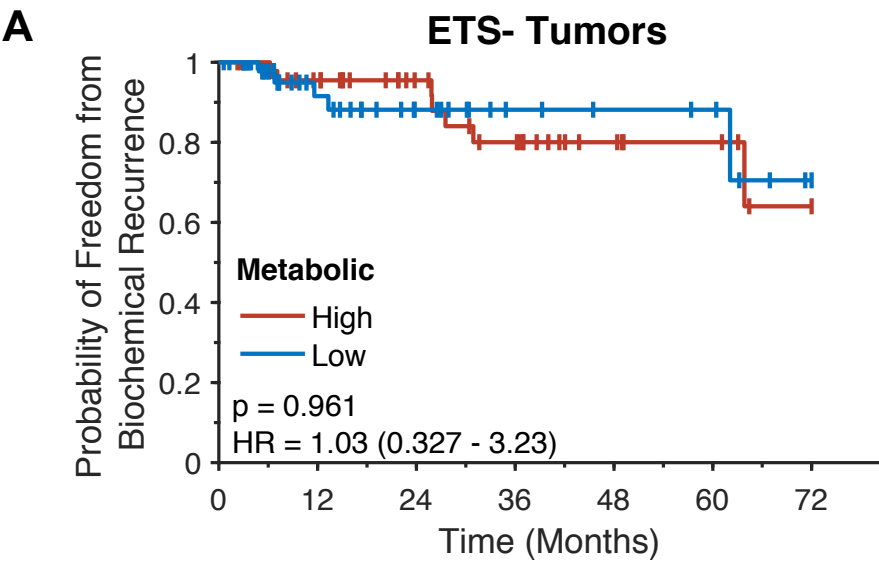

|             |    |    |    |    |    |   |   |
|-------------|----|----|----|----|----|---|---|
| <b>High</b> | 54 | 37 | 26 | 19 | 10 | 7 | 3 |
| <b>Low</b>  | 54 | 27 | 17 | 10 | 7  | 6 | 1 |

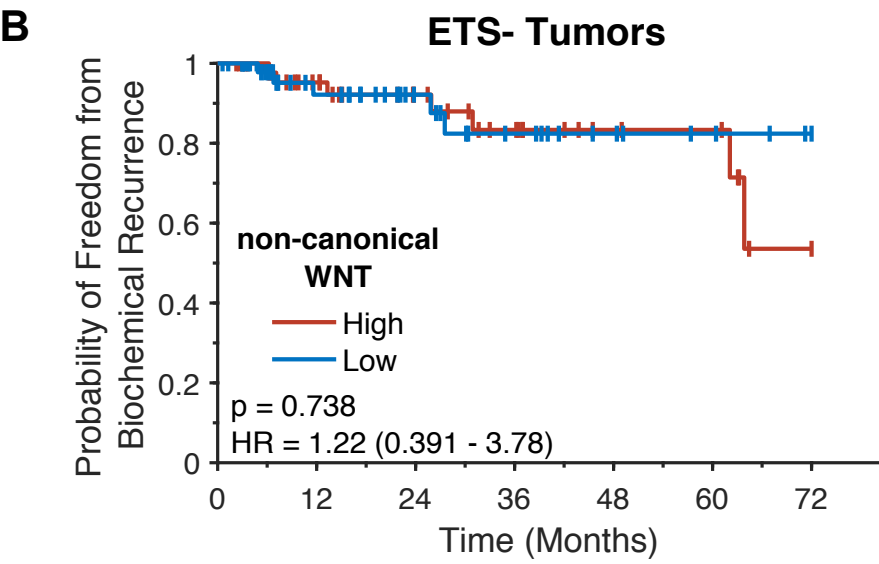

|             |    |    |    |    |   |   |   |
|-------------|----|----|----|----|---|---|---|
| <b>High</b> | 54 | 33 | 23 | 16 | 9 | 8 | 2 |
| <b>low</b>  | 54 | 31 | 20 | 13 | 8 | 5 | 2 |

Supplement: Supplementary file 5 — Supplementary Figure S4 [file 41391_2018_103_MOESM5_ESM.pdf]
