# Supplementary material for: Distinct transcriptional repertoire of the androgen receptor in ETS fusion-negative prostate cancer
Source: Prostate Cancer Prostatic Dis. 2018 Oct 26;22(2):292–302. doi: 10.1038/s41391-018-0103-4 (PMC6760558; doi:10.1038/s41391-018-0103-4)
Supplement: Supplementary file 6 — Table S1 [file 41391_2018_103_MOESM6_ESM.docx]

**Table S1: Clinical, demographic, and pathological characteristics - TCGA and GRID cohorts**

|  | **TCGA, n = 333** | | | | **GRID Data, n = 635** | | | |
| --- | --- | --- | --- | --- | --- | --- | --- | --- |
| **Clinico-Pathological Characteristics** | **ETS + (N = 198)** | | **ETS - (N = 135)** | | **ETS + (N = 326)** | | **ETS - (N = 309)** | |
| **Race** |  |  |  |  |  |  |  |  |
| AAM | 9 (4.5) | | 13 (9.6) | | 32 (9.8) | | 95 (30.7) | |
| EAM | 106 (53.5) | | 57 (42.2) | | 294 (90.2) | | 214 (69.3) | |
| Unk | 83 (41.9) | | 65 (48.1) | |  | - |  | - |
| **ETS Status by race** |  |  |  |  |  |  |  |  |
| Race (AAM\|EAM) | 9 | 106 | 13 | 57 | 32 | 294 | 95 | 214 |
| Proportion by Race | 40.9 | 65.0 | 59.1 | 35.0 | 25.2 | 57.9 | 74.8 | 42.1 |
| **Pathology Gleason Score** |  |  |  |  |  |  |  |  |
| ≤ 3+3 | 35 (17.7) | | 30 (22.2) | | 38 (11.7) | | 20 (6.5) | |
| 3+4 | 60 (30.3) | | 42 (31.1) | | 156 (47.8) | | 132 (42.7) | |
| 4+3 | 53 (26.8) | | 25 (18.5) | | 56 (17.2) | | 59 (19.1) | |
| ≥ 8 | 50 (25.2) | | 38 (28.1) | | 76 (23.3) | | 98 (31.7) | |
| **Prostate Specific Antigen (ng/ml)** |  | |  |  |  |  |  |  |
| ≤ 6 | 43 (21.7) | | 25 (18.5) | | 125 (38.3) | | 99 (32.0) | |
| > 6 - 10 | 41 (20.7) | | 19 (14.1) | | 103 (31.6) | | 88 (28.5) | |
| > 10 - 20 | 23 (11.6) | | 13 (9.6) | | 86 (26.4) | | 93 (30.1) | |
| > 20 | 9 (4.5) | | 14 (10.4) | | 12 (3.7) | | 29 (9.4) | |
| Unk | 82 (41.4) | | 64 (47.4) | |  |  |  |  |
| **Surgical Margins** |  | |  |  |  |  |  |  |
| Present | 38 (19.2) | | 31 (23.0) | | 168 (51.5) | | 156 (50.5) | |
| Absent | 122 (61.6) | | 77 (57.0) | | 158 (48.5) | | 153 (49.5) | |
| Unk | 38 (19.2) | | 27 (20.0) | |  |  |  |  |
| **Extracapsular Extension** |  | |  |  |  |  |  |  |
| Yes | - | |  | - | 225 (69.0) | | 177 (57.3) | |
| No | - | |  | - | 101 (31.0) | | 132 (42.7) | |
| **Lymph Node Invasion** |  | |  |  |  |  |  |  |
| Yes | - | |  | - | 24 (7.4) | | 16 (5.2) | |
| No | - | |  | - | 302 (92.6) | | 293 (94.8) | |
| **Seminal Vesical Invasion** |  | |  |  |  |  |  |  |
| Yes | - | |  | - | 71 (21.8) | | 63 (20.4) | |
| No | - | |  | - | 255 (78.2) | | 246 (79.6) | |
| **CAPRA-S Score** |  | |  |  |  |  |  |  |
| 0 - 2 | - | |  | - | 50 (15.3) | | 29 (9.4) | |
| 3 - 5 | - | |  | - | 176 (54.0) | | 175 (56.6) | |
| 6 - 12 | - | |  | - | 100 (60.7) | | 105 (34.0) | |
| **Age at Diagnosis (Years)** |  | |  |  |  |  |  |  |
| ≤ 50 | 18 (9.1) | | 8 (5.9) | | 15 (4.6) | | 21 (6.8) | |
| > 50 - 65 | 104 (52.5) | | 66 (48.9) | | 245 (75.1) | | 210 (68.0) | |
| > 65 | 44 (22.2) | | 39 (28.4) | | 66 (20.2) | | 78 (25.2) | |
| Unk | 32 (16.2) | | 22 (16.3) | | - | | - | |

**Abbreviation:** CAPRA-S, Cancer of the prostate risk assessment; AAM, African American
