# Supplementary material for: Distinct transcriptional repertoire of the androgen receptor in ETS fusion-negative prostate cancer
Source: Prostate Cancer Prostatic Dis. 2018 Oct 26;22(2):292–302. doi: 10.1038/s41391-018-0103-4 (PMC6760558; doi:10.1038/s41391-018-0103-4)
Supplement: Supplementary file 10 — Table S5 [file 41391_2018_103_MOESM10_ESM.docx]

**Supplementary Table S5: ETS dependent PCa AR target genes enriched for specific biological pathways**

| **Category** | **ETS status** | ***Gene Symbol** | ***Enriched Pathways** |
| --- | --- | --- | --- |
| **Group 1** | **ETS- up** | ***ALOX15B, ABAT, ABHD2, IGFBP2, CYP2J2, ACAD8, GNMT, SLC2A12, NANS, SMS, SLC45A3, RHOU, INPP4B, EFNB2, CAMKK2****, MESP1, MAP2, MYBPC1, BBS4, RLN1, NCAPD3, COL12A1, KLK4, AFF3, FECH, STXBP6, CNTNAP2, H2AFJ, TMPRSS2, ENDOD1, C3orf14, GREB1* | ***Metabolic processes and non-canonical WNT pathway*** |
| **Group 2** | **ETS- Dn** | ***SORL1, GNPTAB, HNF1B, PHLDB2, ITPR1, PDE8B, C8orf4, SEPT9****, ACSL5, B3GNT5, FAM135A, TRIM2, FRK, GP2* | ***Cellular differentiation*** |
| **Group 3** | **ETS+ UP** | ***KCNC2, ITPR3, KCNH8, KCNN2, KCNG3, GRIN3A, SLC10A7, NCALD, SLC26A2, STEAP4, ATP11A, TMC5, PEX10, GUCY1A3, PDE9A, STK39, GHR, MYO6, ZNF385B, CHN2, NKAIN1, F5,*** *CHRM3, REXO2, NUDT11, ERG, OCLN, TLE1, HLA-DMB, UGDH, PXDN, HSD17B11, KIAA1244, KHDRBS3, TMEM2, FAM198B, RHPN2, C11orf92, PPYR1* | ***Signal transduction and ion transport processes*** |
| **Group 4** | **ETS+ Dn** | ***WNT5A, SEMA3E, NBL1, HSPB1, PAK1IP1, AZGP1, GADD45B,*** *RAB27A, TNFAIP8, MAPK4, DBI, NOSTRIN, NAT1, PPFIBP2, LEPREL1* | ***Negative regulators of chemotaxis and cellular motility*** |
| **Group 5** | **ETS-/ETS+ UP** | *ATP8A2, ALDH1A3, GABRB3, SDK1, GCNT1, DLX1, SPOCK1, ADAM2, SPON2, BICD1, SH3RF1, CACNA1D, RAB3B, TOX3, DNAH8, RALGAPA2, TDRD1, SLC4A4, NFE2L3, MCCC2, GDPD1, DNASE2B, C2orf72, LUZP2, ANKRD34B, SAMD5, CHDH* | ***Non-specific*** |

* Bolded gene symbols indicate the specific genes that are enriched within the corresponding pathways
